# Supplementary material for: Theta-gamma phase amplitude coupling serves as a marker of social cognition and visual working memory deficits in individuals with elevated autistic traits
Source: Commun Psychol. 2026 Jan 14;4:25. doi: 10.1038/s44271-025-00392-6 (PMC12881457; doi:10.1038/s44271-025-00392-6)
Supplement: Supplementary file 2 — Supplementary Information [file 44271_2025_392_MOESM2_ESM.pdf]

Supplementary Information

Theta-gamma phase amplitude coupling serves as a marker of social cognition and visual working memory deficits in individuals with elevated autistic traits

Elisabeth V. C. Friedrich[1,2,3*], Yannik Hilla[3,4*], Elisabeth F. Sterner[2,5], Simon S. Ostermeier[2], Larissa Behnke[3,6], Paul Sauseng[3,6]

[1] Faculty of Psychology, University of Sustainability Vienna – Charlotte Fresenius Privatuniversität, Vienna, Austria

[2] Department of Psychology, Research Unit Biological Psychology, Ludwig-Maximilians-Universität München, Munich, Germany

[3] Department of Psychology, Neuropsychology and Cognitive Neuroscience Unit, University of Zurich, Zurich, Switzerland

[4] Department of Human Sciences, Institute of Psychology, University of the Bundeswehr Munich, Neubiberg, Germany

[5] Department of Diagnostic and Interventional Neuroradiology, School of Medicine, Technical University of Munich, Munich, Germany

[6] Neuroscience Center Zurich, University of Zurich, Zurich, Switzerland

***Corresponding authors:**

elisabeth.friedrich-higgs@uni-sustainability.at

yannik.hilla@psychologie.uzh.ch

Supplementary Table 1. Regions of Interest (ROIs).

The x, y, z coordinates are indicated according to the Montreal Neurological Institute (MNI). We based the coordinates on Meyer et al. (2015, 2012), Meyer and Collier (2020) and Todd and Marois (2004). If the indicated literature reported the coordinates in Talairach coordinates, we transferred them to MNI with SLORETA software (Standardized Low Resolution Electromagnetic Tomography; sLORETA v20190617; Pascual-Marqui, 2002; Pascual-Marqui, 2007).

(A) We defined the dorsomedial prefrontal cortex (DMPFC) as frontal region of interest (ROI), from which we extracted the frontal-midline theta (FM-theta) phase. **(B)** We defined 11 posterior ROIs, from which we extracted gamma amplitude values. The ROIs written in purple were associated with social working memory in the cited studies. The ROIs written in blue were found to be active in non-social tasks or responsible for general load effects in working memory processes.

**ROI name**	**ROI abbreviation**	**X (MNI)**	**Y (MNI)**	**Z (MNI)**	**Reference**
**(A)**					
dorsomedial prefrontal cortex	DMPFC	12	29	31	Meyer et al. (2012)
		-12	38	49	Meyer et al. (2012)
		15	39	54	Meyer et al. (2015)
		6	54	24	Meyer et al. (2015)
		12	66	15	Meyer et al. (2015)
		-9	54	39	Meyer et al. (2015)
		12	36	54	Meyer and Collier (2020)
		-6	34	56	Meyer and Collier (2020)
		-4	44	48	Meyer and Collier (2020)
		-8	54	36	Meyer and Collier (2020)
		14	38	54	Meyer and Collier (2020)
		-10	58	26	Meyer and Collier (2020)
**(B)**					
left temporal pole	lTP	-50	10	-32	Meyer and Collier (2020)
		-62	-10	-22	Meyer and Collier (2020)
		-60	-2	-24	Meyer and Collier (2020)
right temporal pole	rTP	48	12	-36	Meyer and Collier (2020)
		52	16	-28	Meyer and Collier (2020)
		62	-6	-18	Meyer and Collier (2020)
left temporo-parietal junction	lTPJ	-42	-70	40	Meyer et al. (2012)
		-52	-64	40	Meyer and Collier (2020)
		-48	-58	30	Meyer and Collier (2020)
		-56	-64	26	Meyer and Collier (2020)
		-54	-66	24	Meyer and Collier (2020)
		-42	-62	24	Meyer and Collier (2020)
		-50	-66	16	Meyer and Collier (2020)

right temporo-parietal junction	rTPJ	42	-54	24	Meyer et al. (2015)
		54	-66	27	Meyer et al. (2015)
		52	-60	44	Meyer and Collier (2020)
left inferior parietal lobe	lIPL	-33	-54	45	Meyer et al. (2015)
right inferior parietal lobe	rIPL	45	-33	45	Meyer et al. (2015)
		33	-66	48	Meyer et al. (2015)
		44	-36	44	Meyer and Collier (2020)
left intraparietal sulcus	lIPS	-22	-69	42	Todd and Marois (2004)
right intraparietal sulcus	rIPS	23	-63	46	Todd and Marois (2004)
medial precuneus/ posterior cingulate cortex	PC/PCC	0	-61	46	Meyer et al. (2012)
		-3	-60	27	Meyer et al. (2015)
		-3	-54	21	Meyer et al. (2015)
		3	-54	36	Meyer et al. (2015)
		-2	-50	28	Meyer and Collier (2020)
		0	-68	36	Meyer and Collier (2020)
left precuneus/ posterior cingulate	lPC/PCC	-10	-60	54	Meyer and Collier (2020)
right precuneus/ posterior cingulate	rPC/PCC	12	-62	58	Meyer and Collier (2020)
		4	-42	46	Meyer and Collier (2020)

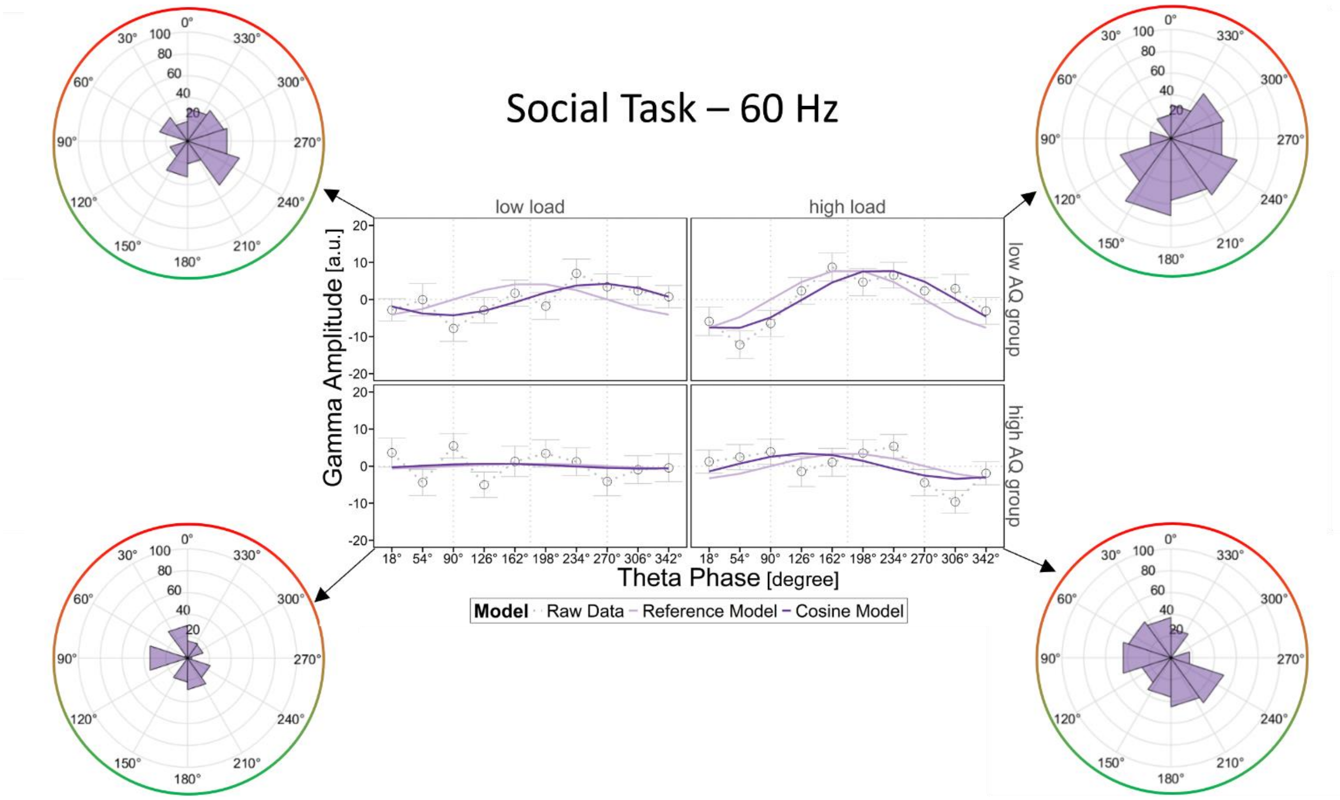

Supplementary Figure 1. DMPFC Phase-Amplitude Coupling in the Social Task. Frontal-midline theta **(**FM-theta) phase was extracted from the dorsomedial prefrontal cortex (DMPFC). 60-Hz posterior gamma amplitude was extracted from 11 posterior regions of interest (ROIs, see Supplementary Table 1). The z-transformed posterior instantaneous gamma amplitude was sorted according to instantaneous FM-theta phase and averaged over all 11 posterior ROIs. In the line charts, the grey dots indicate the empirical z-transformed and sorted 60-Hz gamma amplitudes, the grey whiskers indicate error bars (i.e., mean +/- standard error (SE). The light purple lines our null-shift reference cosine model (simulating that strongest gamma amplitudes were locked in the trough of FM-theta phase) and the dark purple lines the cosine model fitted to our empirical data. In the circular plots, z-transformed sorted gamma amplitudes (purple) are displayed as percentage of the signal, FM-theta peak (0°) is indicated in red and FM-theta trough (180°) in green.
In the social task in the here displayed 60-Hz frequency band, the phase-amplitude coupling was similar but slightly weaker than in the 70-Hz frequency band, described in the main manuscript and illustrated in Figure 4C.

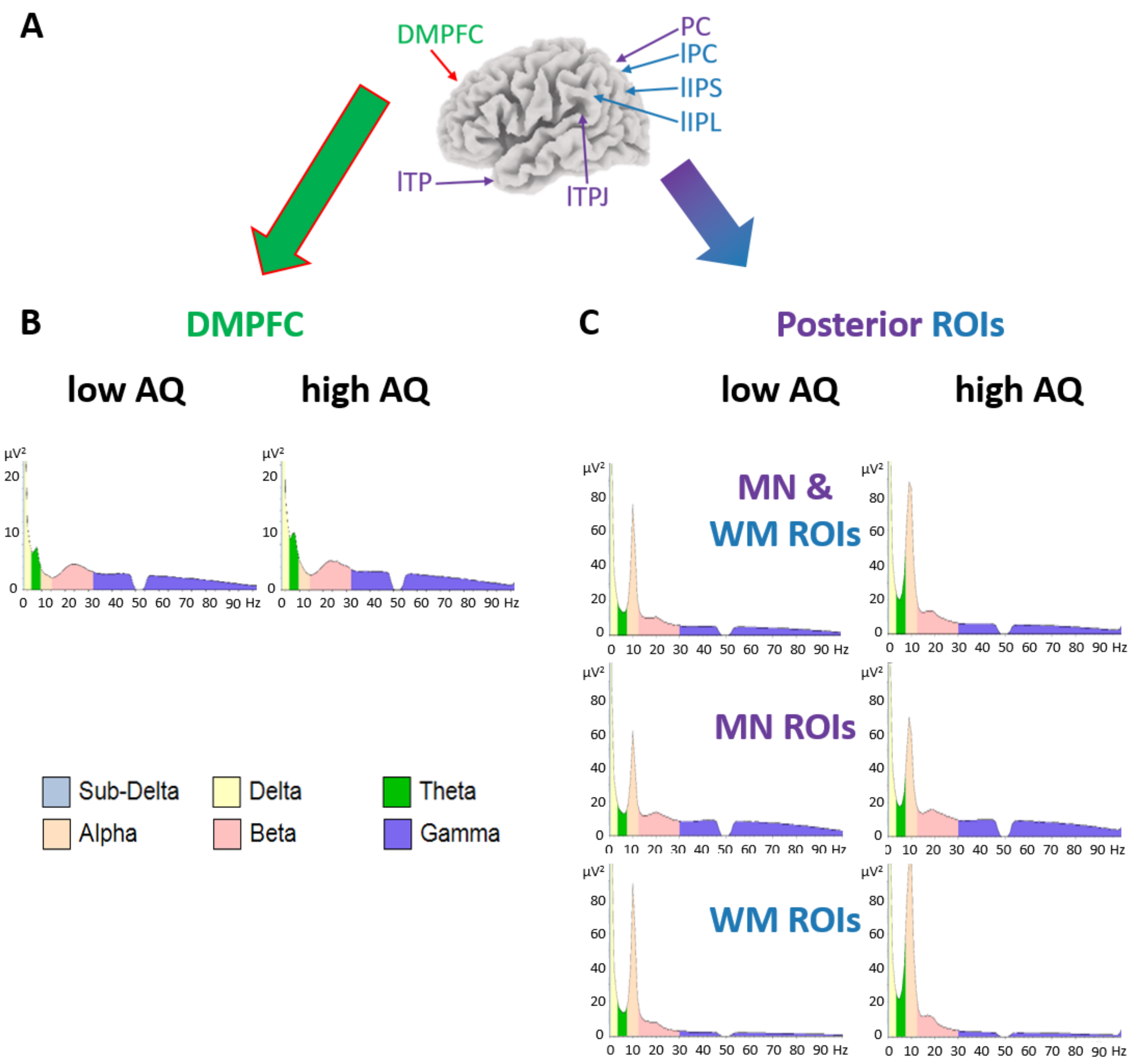

Supplementary Figure 2. Power Spectra.

(A) Data was extracted from one frontal ROI (i.e., dorsomedial prefrontal cortex (DMPFC, green/red) and eleven posterior ROIs. Five of these posterior ROIs were reported to be active during social working memory tasks (left/right temporal pole (l/rTP), left/right temporo-parietal junction (l/rTPJ), medial precuneus (PC)) and match with regions from the mentalizing network (MN, in purple; (Meyer et al., 2015, 2012; Meyer and Collier, 2020). Six of these regions were found to be active in nonsocial tasks or responsible for general load effects in working memory processes (left/right precuneus/posterior cingulate cortex (l/rPC), left/right inferior parietal lobe (l/rIPL), left/right intraparietal sulcus (l/rIPS)) and considered typical working memory regions (WM, in blue; Meyer et al., 2015; Meyer and Collier, 2020; Todd and Marois, 2004). The arrows show the approximate left and medial ROIs, for all coordinates see Supplementary Table 1.

(B) Power (μV^2) averaged over the first 2.5-s of the manipulation period (y- axis) are shown for the freuqency range of 1-100 Hz (x-axis) with a 1 Hz resolution for the DMPFC, seperatley for

the low (left column) and high (right colum) autistic-traits (AQ) groups. A clear theta peak (green) is visible in the power spectra at the DMPFC for both groups.

(C) Power (μV^2) averaged over the first 2.5-s of the manipulation period (y- axis) are shown for the freuqency range of 1-100 Hz (x-axis) with a 1 Hz resolution, seperatley for the low (left column) and high (right colum) autistic-traits (AQ) groups. In the first row, power was averaged over all eleven posterior ROIs (MN & WM ROIs). In the second row, power was averaged over the five posterior ROIs associated with the mentalizing network (MN ROIs). In the third row, power was averaged over the six posterior ROIs associated with the working memory system (WM ROIs). No theta peaks are visible in the power spectra at the posterior ROIs.

Supplementary Table 2. Statistical Amplitude Analysis. (A) We calculated repeated-measures ANOVA for FM-theta amplitude at the DMPFC with the within-subject factor load and between-subject factor autistic-traits (AQ) group separately for the tasks. As expected, FM-theta amplitude was higher in the high than low load condition in all tasks. There was no statistically significant difference in FM-theta amplitude between the autistic-traits groups and no significant interaction in any task. **(B)** We calculated repeated-measures ANOVA for gamma amplitude separately for the tasks and the center frequencies of 60 Hz and 70 Hz with the within-subject factor load and posterior ROIs and the between-subject factor autistic-traits (AQ) group. There was no main effect in load or autistic-traits groups and no significant interaction between these factors or involving posterior ROIs in any task. The main effect ROIs became significant in all tasks and frequency bands, however, this effect is trivial and to be expected. Importantly, it was not systematically modulated by load or autistic-traits group. Moreover, the z-transformation of gamma amplitudes for the phase-amplitude coupling eliminated this effect. *(abb.: partial η^2 = $p\eta^2$)*

**(A) FM-theta (4-7 Hz) amplitude at DMPFC**		
**4-7 Hz**		
verbal	load	$F(1, 96) = 16.418$, $p < 0.001$, partial $\eta^2 = 0.146$
	AQ group	$F(1, 96) = 2.386$, $p = 0.126$, partial $\eta^2 = 0.024$
	load x AQ group	$F(1, 96) = 0.043$, $p = 0.836$, partial $\eta^2 = 0.000$
social	load	$F(1, 96) = 4.808$, $p = 0.031$, partial $\eta^2 = 0.048$
	AQ group	$F(1, 96) = 3.506$, $p = 0.064$, partial $\eta^2 = 0.035$
	load x AQ group	$F(1, 96) = 0.260$, $p = 0.611$, partial $\eta^2 = 0.003$
visual	load	$F(1, 96) = 24.934$, $p < 0.001$, partial $\eta^2 = 0.206$
	AQ group	$F(1, 96) = 2.616$, $p = 0.109$, partial $\eta^2 = 0.027$
	load x AQ group	$F(1, 96) = 0.054$, $p = 0.817$, partial $\eta^2 = 0.001$
**(B) Gamma amplitude at posterior ROIs**		
**60 Hz**		
social	load	$F(1, 96) = 0.659$, $p = 0.419$, partial $\eta^2 = 0.007$
	AQ group	$F(1, 96) = 0.611$, $p = 0.436$, partial $\eta^2 = 0.006$
	load x AQ group	$F(1, 96) = 0.003$, $p = 0.953$, partial $\eta^2 = 0.000$
	ROIs	$F(2.145, 205.938) = 109.109$, $p < 0.001$, $p\eta^2 = 0.532$
	load x ROIs	$F(2.185, 209.765) = 1.518$, $p = 0.220$, $p\eta^2 = 0.016$
	AQ group x ROIs	$F(2.145, 205.938) = 0.295$, $p = 0.760$, $p\eta^2 = 0.003$
	load x AQ group x ROIs	$F(2.185, 209.765) = 0.592$, $p = 0.569$, $p\eta^2 = 0.006$
**70 Hz**		
social	load	$F(1, 96) = 0.721$, $p = 0.398$, partial $\eta^2 = 0.007$
	AQ group	$F(1, 96) = 0.584$, $p = 0.447$, partial $\eta^2 = 0.006$
	load x AQ group	$F(1, 96) = 0.001$, $p = 0.970$, partial $\eta^2 = 0.000$
	ROIs	$F(2.146, 206.060) = 108.085$, $p < 0.001$, $p\eta^2 = 0.530$
	load x ROIs	$F(2.153, 206.719) = 1.562$, $p = 0.211$, $p\eta^2 = 0.016$
	AQ group x ROIs	$F(2.146, 206.060) = 0.268$, $p = 0.780$, $p\eta^2 = 0.003$
	load x AQ group x ROIs	$F(2.153, 206.719) = 0.660$, $p = 0.529$, $p\eta^2 = 0.007$
**70 Hz**		
visual	load	$F(1, 96) = 2.055$, $p = 0.155$, partial $\eta^2 = 0.021$
	AQ group	$F(1, 96) = 2.373$, $p = 0.127$, partial $\eta^2 = 0.024$
	load x AQ group	$F(1, 96) = 0.341$, $p = 0.561$, partial $\eta^2 = 0.004$
	ROIs	$F(1.925, 184.757) = 121.715$, $p < 0.001$, $p\eta^2 = 0.559$
	load x ROIs	$F(2.392, 229.638) = 0.719$, $p = 0.512$, $p\eta^2 = 0.007$
	AQ group x ROIs	$F(1.925, 184.757) = 0.875$, $p = 0.415$, $p\eta^2 = 0.009$
	load x AQ group x ROIs	$F(2.392, 229.638) = 0.673$, $p = 0.537$, $p\eta^2 = 0.007$

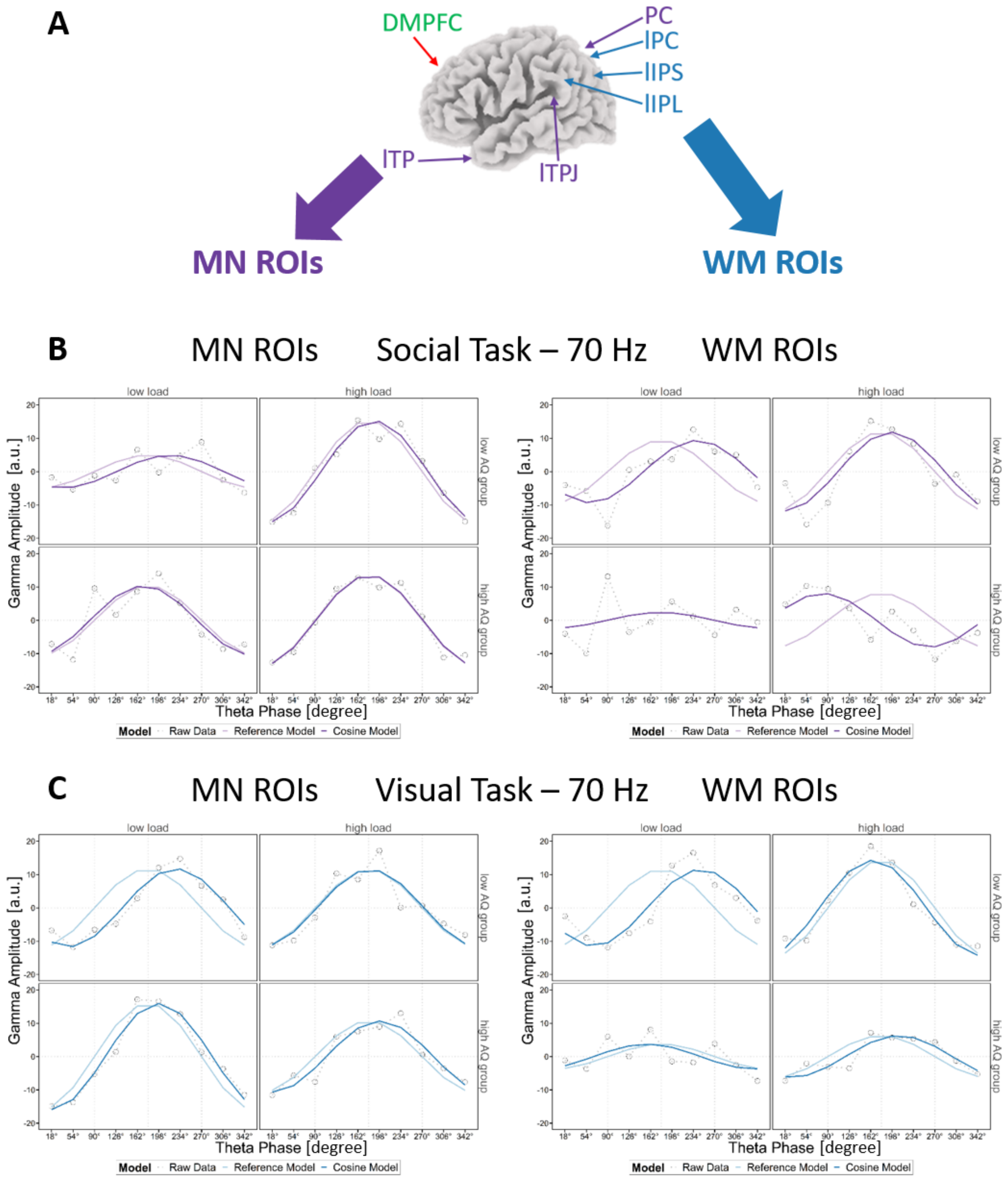

Supplementary Figure 3. DMPFC Phase-Amplitude Coupling separately for posterior ROIs associated with the mentalizing network (MN) and working memory system (WM).

(A) FM-theta phase was extracted from the dorsomedial prefrontal cortex (DMPFC; green/red). 70-Hz posterior gamma amplitude was extracted from 11 posterior regions of interest. Five of these posterior ROIs were reported to be active during social working memory tasks (left/right temporal pole (l/rTP), left/right temporo-parietal junction (l/rTPJ), medial precuneus (PC)) and match with regions from the mentalizing network (MN, in purple; (Meyer et al., 2015, 2012; Meyer and Collier, 2020). Six of these regions were found to be active in non-social tasks or

responsible for general load effects in working memory processes (left/right precuneus/posterior cingulate cortex (l/rPC), left/right inferior parietal lobe (l/rIPL), left/right intraparietal sulcus (l/rIPS)) and considered typical working memory regions (WM, in blue; Meyer et al., 2015; Meyer and Collier, 2020; Todd and Marois, 2004). The arrows show the approximate left and medial ROIs, for all coordinates see Supplementary Table 1.

(B) and **(C)** The z-transformed posterior instantaneous gamma amplitude was sorted according to instantaneous FM-theta phase and averaged either over the ROIs associated with the mentalizing network (left) or the working memory system (right).
In the single line charts, the grey dots indicate the empirical z-transformed and sorted 70-Hz gamma amplitudes, the light purple (i.e., social task (B)) or blue (i.e., visual task (C)) lines our null-shift reference cosine model (simulating that strongest gamma amplitudes were locked in the trough of FM-theta phase) and the dark purple (i.e., social task (B)) or blue (i.e., visual task (C)) lines the cosine model fitted to our empirical data.
In the social (B) and visual (C) tasks, in the low autistic-traits (AQ) group (top rows), the same pattern emerged in the phase-amplitude coupling with mentalizing and working memory regions: In the low load condition, strongest gamma amplitude was shifted towards 270°, while the maximal gamma amplitude was locked to the FM-theta phase in the high load condition. In the high autistic-traits (AQ) group (bottom rows), the load-dependent phase-amplitude coupling (as obtained for individuals with low autistic traits), could not be systematically found.

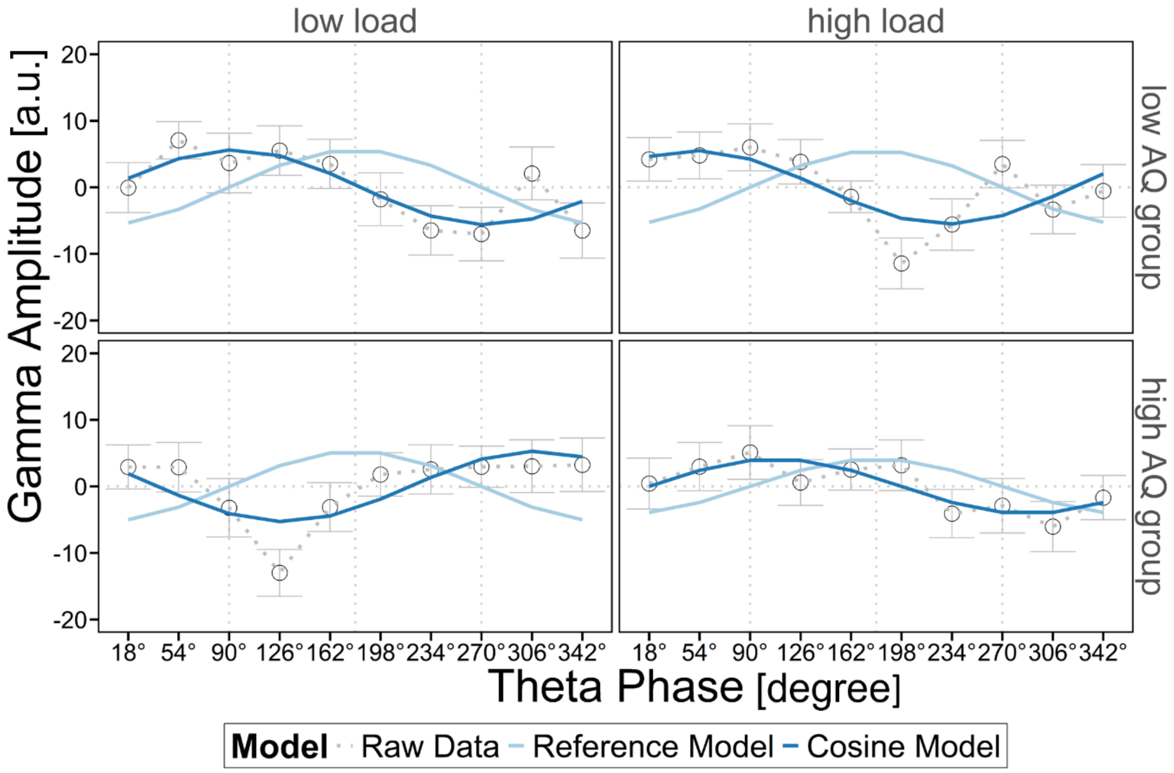

Supplementary Figure 4. Control Analysis with shifted data for DMPFC Phase-Amplitude Coupling in the Visual Task (70-Hz gamma amplitude) to control for evoked effects.

The FM-theta phase values and gamma amplitude were shifted by one trial, so that theta phase from trial 1 was now coupled with gamma amplitude from the last trial and theta phase from trial 2 was coupled with gamma amplitude from the first trial. This way only stimulus evoked effects remained in the data without any induced effects surviving the re-alignment. We then calculated the same phase-amplitude coupling values, regression models, and cosine fitting models. The results indicated a significant interaction in the regression models in the 70-Hz gamma amplitude between FM-theta phase segments, load and autistic-traits groups in the visual task. However, in contrast to the results from the original analysis, the data did not fit significantly better to a cosine model than an intercept model in the low autistic-traits group ($AICc_{low\ load}$ = -0.07, $MAEr_{low\ load}$ = 2.91; $AICc_{high\ load}$ = -2.39, $MAEr_{high\ load}$ = 4.01; please note: AICc >2 indicates that the data fit significantly better to a cosine than an intercept model). Also in the high autistic-traits group in the low load condition, the data did not fit significantly better to a cosine model than an intercept model (AICc = -1.76, MAEr = 3.30). Only in the high autistic-traits group in the high load condition, the data fit significantly better to a cosine model than an intercept model (AICc = 2.62, MAEr = 5.11). However, the modulation showed the reversed coupling pattern than in the main analyses (see Figure 4). These results indicate that the findings from the main analyses cannot be explained by mere stimulus evoked effects. Please note: The grey dots indicate the empirical z-transformed and sorted 70-Hz gamma amplitudes, the grey whiskers indicate error bars (i.e., mean +/- standard error (SE). The light blue lines our null-shift reference cosine model (simulating that strongest gamma amplitudes were locked in the trough of FM-theta phase) and the dark blue lines the cosine model fitted to our empirical data.

Supplementary Table 3. Left Dorsolateral Prefrontal Cortex (lDLPFC). The x, y, z coordinates are indicated for the left dorsolateral prefrontal cortex according to the Montreal Neurological Institute (MNI). We based the coordinates on Meyer et al. (2015, 2012).

ROI name	ROI abbreviation	X (MNI)	Y (MNI)	Z (MNI)	Reference
left dorsolateral	lDLPFC	-45	17	28	Meyer et al. (2012)
prefrontal		-45	30	24	Meyer et al. (2015)
cortex		-51	9	39	Meyer et al. (2015)

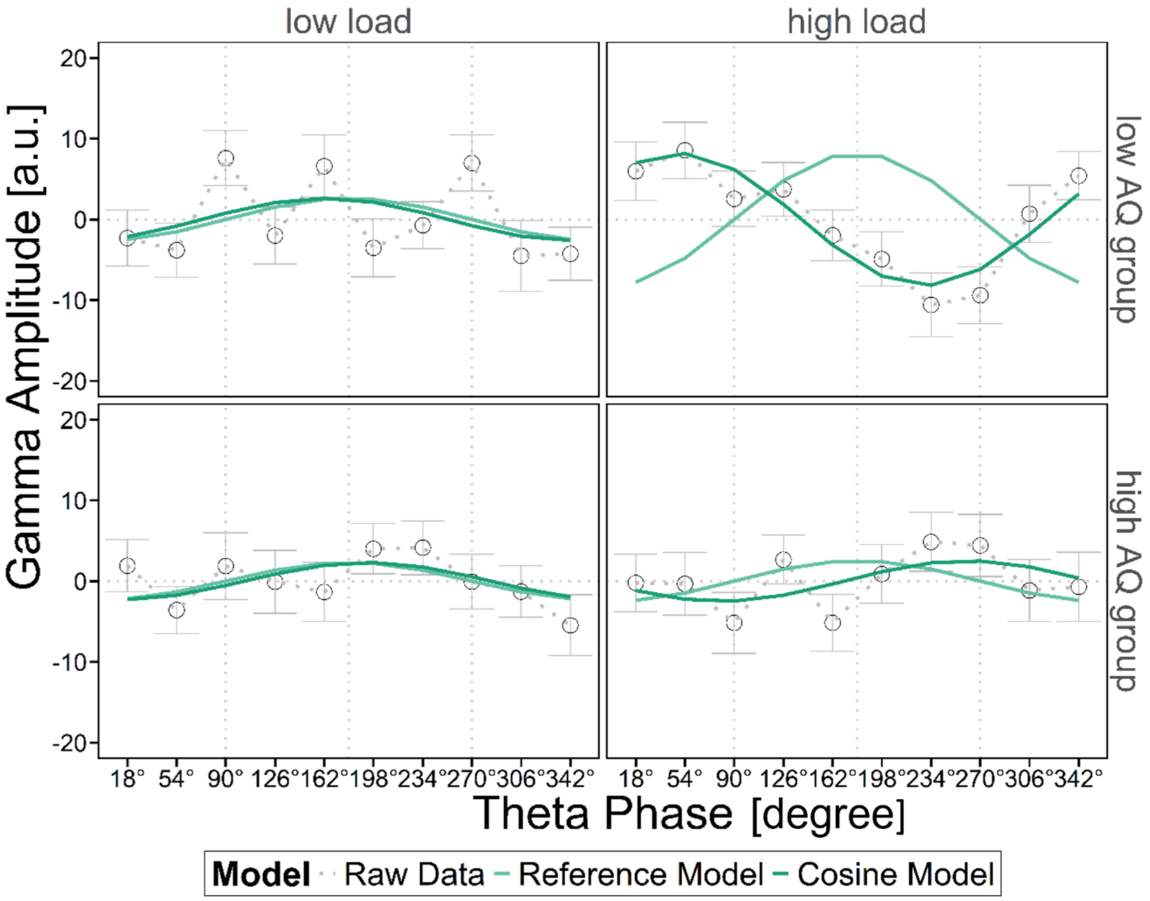

Supplementary Figure 5. Left DLPFC Phase-Amplitude Coupling in the Verbal Task. Theta phase was extracted from the left dorsolateral prefrontal cortex (lDLPFC). 50-Hz posterior gamma amplitude was extracted from 11 posterior regions of interest (ROIs, see Supplementary Table 1). The z-transformed posterior instantaneous gamma amplitude was sorted according to instantaneous lDLPFC-theta phase and averaged over all 11 posterior ROIs. In the line charts, the grey dots indicate the empirical z-transformed and sorted 50-Hz gamma amplitudes, the grey whiskers indicate error bars (i.e., mean +/- standard error (SE). The light turquoise lines our null-shift reference cosine model (simulating that strongest gamma amplitudes were locked in the trough of theta phase) and the dark turquoise lines the cosine model fitted to our empirical data.

In the verbal task in the 50-Hz frequency band in the low autistic-traits (AQ) group (top row) in the low load condition, the intercept model described the data better than a cosine model (AICc = -8.65). In the high load condition, the cosine model described the data significantly better than the intercept model (AICc = 9.90) but there was a significant phase shift, indicating that gamma amplitude was rather locked closer to the peak than the theta trough. In the high autistic-traits group (bottom row), the intercept model described the data better than the cosine model in both load conditions ($AICc_{low\ load}$ = -6.65; $AICc_{high\ load}$ = -6.74).

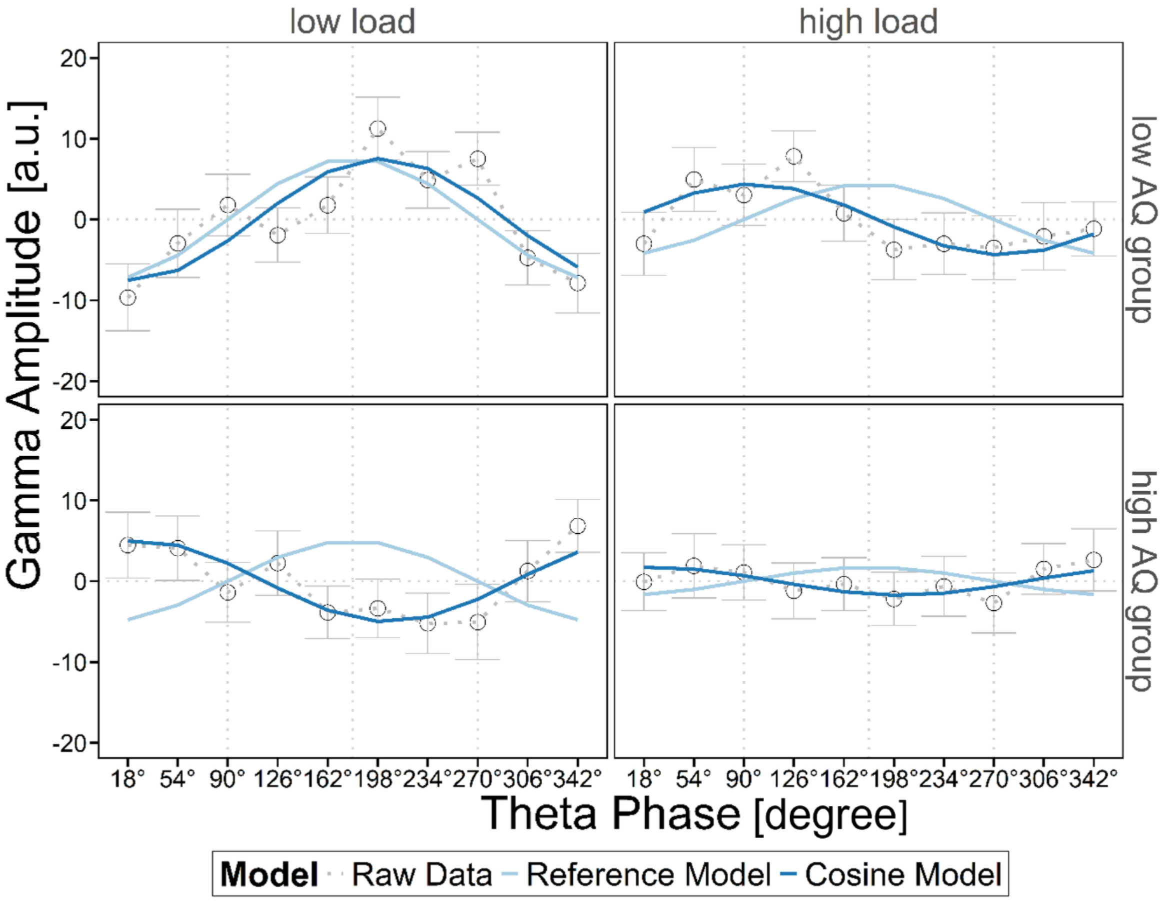

Supplementary Figure 6. Left DLPFC Phase-Amplitude Coupling in the Visual Task. Theta phase was extracted from the left dorsolateral prefrontal cortex (lDLPFC). 50-Hz posterior gamma amplitude was extracted from 11 posterior regions of interest (ROIs, see Supplementary Table 1). The z-transformed posterior instantaneous gamma amplitude was sorted according to instantaneous lDLPFC-theta phase and averaged over all 11 posterior ROIs. In the line charts, the grey dots indicate the empirical z-transformed and sorted 50-Hz gamma amplitudes, the grey whiskers indicate error bars (i.e., mean +/- standard error (SE). The light blue lines our null-shift reference cosine model (simulating that strongest gamma amplitudes were locked in the trough of theta phase) and the dark blue lines the cosine model fitted to our empirical data.

In the visual task in the 50-Hz frequency band in the low autistic-traits (AQ) group (top row), the cosine model did not significantly describe the data better than the intercept model ($AICc_{low\ load}$ = 1.92; $AICc_{high\ load}$ = 0.56). In the high autistic-traits group (bottom row) in the low load condition, the cosine model described the data significantly better than the intercept model (AICc = 3.10) and there was a significant phase shift, indicating that gamma amplitude was rather locked closer to the peak than the theta trough. In the high autistic-traits group in the high load condition, the intercept model described the data better than the cosine model (AICc = -2.71).

Supplementary Table 4. Descriptive statistics. The mean and standard deviation (SD) for accuracy (%) and reaction time (ms) are displayed for each participant group, task and load condition.

**Group**	**Low Autistic-Traits Group**						**High Autistic-Traits Group**					
**Task**	**social**		**visual**		**verbal**		**social**		**visual**		**verbal**	
**Load**	**low**	**high**	**low**	**high**	**low**	**high**	**low**	**high**	**low**	**high**	**low**	**high**
**Accuracy**												
mean	82.2	73.6	93.8	81.6	91.3	79.8	81.5	70.1	94.6	82.6	94.6	81.9
SD	7.41	10.0	4.69	9.17	10.8	12.3	8.97	10.1	4.63	7.81	6.86	9.19
**Reaction Time**												
mean	1108	1584	1182	1655	1143	1673	1117	1675	1183	1743	1167	1767
SD	230	285	256	304	229	370	190	333	208	327	220	335

Supplementary Table 5. Social Questionnaire Re-test Reliability. We evaluated the re-test reliability of our social questionnaire (i.e., individualized questionnaire of the screening to build the trials for the social task in the EEG experiment) in a separate study. We asked 63 participants to fill out the social questionnaire online twice, evaluating the same contact persons in both sessions. Four participants had to be excluded due to invalid data. The final sample included 35 participants with low autistic-traits and 24 participants with high autistic-traits (AQ: M = 27.08, SD = 6.06) based on the Autism Spectrum Quotient (AQ; Baron-Cohen et al., 2001: M = 13.74, SD = 3.73). The low autistic-traits group included 30 women and 5 men, who were on average 22.34 years old (SD = 4.24) and had 14.89 days (SD = 4.53) between the two sessions. The high autistic-traits group included 18 women, 4 men, and 2 gender-diverse participants, who were on average 22.88 years old (SD = 4.70) and had 15.75 days (SD = 4.22) between the two sessions.

The data from the social questionnaire was analyzed in the same way as we did for the social task for the EEG recordings: We only included traits for which participants had ranked their contacts ≥ 15 points apart from one another. Then, we calculated repeated-measures correlation (repeated measures being the 10 contact persons) separately for every trait with > 15 participants in each autistic-traits group between sessions 1 and 2 in R (version 4.2.2, R Studio 2022.12.0; R Core Team, 2022). The value of the repeated measures correlation coefficient (r), the 95% confidence interval (CI) for the repeated measures coefficient and the actual number of participants are displayed for all 32 traits, which fulfilled our criteria (i.e., traits for which at least 15 participants have ranked their contact persons ≥ 15 points apart from one another).

The low autistic-traits group reached a mean re-test reliability of r = 0.75 (range between 0.61 and 0.86). The high autistic-traits group reached a mean re-test reliability of r = 0.73 (range between 0.59 and 0.85). All confidence intervals between the low and high autistic-traits groups overlap, except for the one of trait number 35 (“reif”/“mature”), indicated with an *.

**Number**	**Trait**	**Low Autistic-Traits Group**			**High Autistic-Traits Group**		
**of Trait**	**(German)**	**r**	**95% CI**	**N**	**r**	**95% CI**	**N**
1	analytisch	0.811	0.766 - 0.848	30	0.746	0.674 - 0.805	20
4	aufbrausend	0.795	0.745 - 0.836	28	0.764	0.696 - 0.819	20
6	beharrlich	0.697	0.614 - 0.765	20	0.646	0.550 - 0.726	19
7	berechnend	0.703	0.631 - 0.764	25	0.664	0.562 - 0.746	16
9	dominant	0.837	0.792 - 0.873	24	0.818	0.761 - 0.862	19
12	eigenwillig	0.775	0.708 - 0.829	19	0.632	0.523 - 0.721	16
14	einfühlsam	0.766	0.703 - 0.817	23	0.809	0.750 - 0.855	19
15	eitel	0.764	0.698 - 0.818	21	0.729	0.655 - 0.790	21
17	empfindlich	0.783	0.727 - 0.829	25	0.797	0.731 - 0.848	17
18	entschlossen	0.733	0.665 - 0.789	24	0.627	0.520 - 0.714	17
19	flexibel	0.614	0.524 - 0.691	24	0.725	0.637 - 0.794	16
20	friedlich	0.816	0.765 - 0.857	23	0.779	0.711 - 0.833	18
21	furchtsam	0.796	0.741 - 0.840	24	0.765	0.688 - 0.825	16
22	gehemmt	0.768	0.707 - 0.817	24	0.742	0.661 - 0.805	17
24	gesellig	0.822	0.771 - 0.862	22	0.827	0.773 - 0.869	19
25	gütig	0.669	0.582 - 0.741	21	0.788	0.718 - 0.843	16
27	impulsiv	0.627	0.532 - 0.706	21	0.724	0.642 - 0.790	18
30	kämpferisch	0.749	0.676 - 0.807	20	0.693	0.598 - 0.769	16

31	kritisch	0.651	0.569 - 0.720	25	0.594	0.484 - 0.685	18
32	launisch	0.783	0.729 - 0.827	27	0.796	0.732 - 0.847	18
34	rational	0.711	0.641 - 0.769	26	0.724	0.642 - 0.790	18
35*	reif	0.861	0.816 - 0.895	19	0.741	0.658 - 0.807	16
36	reizbar	0.794	0.742 - 0.836	27	0.778	0.711 - 0.831	19
37	sachlich	0.751	0.685 - 0.805	23	0.761	0.686 - 0.821	17
38	schüchtern	0.756	0.692 - 0.808	24	0.849	0.798 - 0.888	17
39	sensibel	0.652	0.562 - 0.727	21	0.699	0.618 - 0.765	21
40	sentimental	0.648	0.561 - 0.720	23	0.666	0.574 - 0.742	19
41	spontan	0.739	0.677 - 0.790	28	0.68	0.584 - 0.756	17
43	stur	0.803	0.748 - 0.848	22	0.727	0.648 - 0.791	19
46	unsicher	0.807	0.756 - 0.848	25	0.728	0.641 - 0.797	16
47	verspielt	0.707	0.638 - 0.764	27	0.707	0.624 - 0.775	19
48	zaghaft	0.737	0.679 - 0.785	32	0.618	0.506 - 0.709	16

Supplementary References

Baron-Cohen, S., Wheelwright, S., Skinner, R., Martin, J., Clubley, E., 2001. The Autism Spectrum Quotient : Evidence from Asperger syndrome/high functioning autism, males and females, scientists and mathematicians. J. Autism Dev. Disord. 31, 5–17. https://doi.org/10.1023/A:1005653411471

Meyer, M.L., Collier, E., 2020. Theory of minds: Managing mental state inferences in working memory is associated with the dorsomedial subsystem of the default network and social integration. Soc. Cogn. Affect. Neurosci. 15, 63–73. https://doi.org/10.1093/scan/nsaa022

Meyer, M.L., Spunt, R.P., Berkman, E.T., Taylor, S.E., Lieberman, M.D., 2012. Evidence for social working memory from a parametric functional MRI study. Proc. Natl. Acad. Sci. 109, 1883–1888. https://doi.org/10.1073/pnas.1121077109

Meyer, M.L., Taylor, S.E., Lieberman, M.D., 2015. Social working memory and its distinctive link to social cognitive ability: An fMRI study. Soc. Cogn. Affect. Neurosci. 10, 1338–1347. https://doi.org/10.1093/scan/nsv065

Pascual-Marqui, R.D., 2007. Discrete, 3D distributed, linear imaging methods of electric neuronal activity. Part 1: exact, zero error localization. arXiv:0710.3341 1–16.

Pascual-Marqui, R.D., 2002. Standardized low resolution brain electromagnetic tomography (sLORETA): technical details. Methods Find. Exp. Clin. Pharmacol. 1–16. https://doi.org/841 [pii]

R Core Team, N. a., 2022. R: A Language and Environment for Statistical Computing. Vienna, Austria. URL https//www.R-project.org.

Todd, J.J., Marois, R., 2004. Capacity limit of visual short-term memory in human posterior parietal cortex. Nature 428, 751–754. https://doi.org/10.1038/nature02466
